# What is an “ArchintorTM?” A paradigm shift in teaching, facilitation, and learning: The impact of different types of coursework expectations on classroom network structures

**DOI:** 10.1371/journal.pone.0288136

**Published:** 2023-07-19

**Authors:** Hannah B. Love, Ellyn M. Dickmann, Ellen R. Fisher

**Affiliations:** 1 Divergent Science LLC, Fort Collins, Colorado, United States of America; 2 Dickmann and Associates LLC, Loveland, Colorado, United States of America; 3 Department of Chemistry and Chemical Biology, University of New Mexico, Albuquerque, New Mexico, United States of America; University of Stavanger: Universitetet i Stavanger, NORWAY

## Abstract

Hypothetically, a student could attend a class, listen to lectures, and pass the class without knowing or interacting with other students. What happens to the network when the classroom expectations change? For example, there is a coursework expectation that students exchange contact information, or the instructor uses collaborative learning practices. Or what if the principal investigator (PI) of a scientific team goes on a sabbatical? This study uses the framework of classrooms because of their relatability across science. We asked how do different instructor coursework expectations change network structures within a classroom or other learning environments? A social network survey was administered at the start and end of the semester (pre- and post-test) in six university sociology classrooms to explore how expectations impacted the communication and learning networks. We found practical changes in course expectations impact the communication and learning networks, suggesting that instructors, facilitators, and others could be the archintor^TM^ (**arch**itect+**in**structor+facilita**tor**) of the network. Understanding that expectations can impact a network’s structure marks a paradigm shift in educational assessment approaches. If the archintor^TM^ has identified the “optimal” network structure, then their task is to design expectations that result in specific interactions that ultimately improve student achievement and success. This work provides recommendations for classroom archintors^TM^ to create the most impactful classroom networks. Future research should extend beyond education and classroom networks and identify the best or desired networks in other areas like public policy, urban planning, and more. If these “optimal” networks were identified, an archintor^TM^ could design a social network to solve wicked problems, manage a crisis, and create social change.

## Introduction

The world is increasingly interconnected, unpredictable, and uncertainty abounds. Between the Covid-19 pandemic, technological seismic shifts, significant climate changes, and unstable geopolitical conditions; educators are faced with the task of educating students for a constant state of change and for jobs that do not exist yet [1]. How do we create stable networks in an unstable and unpredictable world [2, 3]? What does the preparation for this look like? How is it practiced? How do you know what is most effective? This study utilizes classroom networks as the foundation for understanding how expectations can shape a network because of the potential for knowledge adoption and applicability across all learning environments [4, 5]. With careful planning and follow-up, most instructors can implement new classroom expectations to shape the network.

Historically, teaching has been understood as the transfer of information from the teacher to the student; learning was the ability to acquire and repeat information, and assessment of learning was based on a student’s ability to repeat information [6]. Today, the mundane memorization of facts and figures historically common in classrooms is losing relevance and is often considered patriarchal. Learning is no longer seen as simply repeating information; it involves intellectual synthesis, understanding, and making practical sense of experience [6, 7]. Sternberg and Grigorenko [8] wrote that successful intelligence “is the use of an integrated set of abilities needed to attain success in life; however, an individual defines it, within his or her sociocultural context” (p. 208). Today instructors are not just teaching, they are creating the opportunity for learning [1]. Further, a growing body of literature has found that learning is a social process and involves networks inside and outside of the classroom [9–14].

Chickering [15] outlined seven best practices for higher education which include: promoting contact between students and faculty, developing reciprocity and cooperation among students, encouraging active learning, and communicating high expectations. Chickering [15] further explained that “There is good evidence that such an environment can be created” (p. 7). Other studies agree with Chickering that there is a constructivist aspect to learning [6, 16, 17], that meaning is created through, “inquiry, knowledge acquisition, and the relationships and conversations among people who learn” [6] (p. 4), and the facilitation of different learning practices produce different learning outcomes [18]. Despite evidence that coursework networks can be socially constructed, and this contributes to student learning, there is little evidence to explain how learning is socially constructed [4, 5]. To determine this, studies need to use social network analysis to examine network structures and determine desired structural outcomes in those networks.

The limited studies on in-person classroom social networks reveal that if these settings have well-connected networks they are associated with better academic performance, a positive emotional climate, and improved group performance [19–21]. To date, most studies analyze data from collaborative on-line courses and student discourses [22–25]. Zhang et al., [17] found that in classrooms where students had a shared vision, trust, and knowledge within high-intensity collaborations, their digital trail in discussion forums created a distributed network pattern wherein all students learned and built knowledge. More recently, Love et al. [10] used social network analysis to reveal that some learning practices such as service-learning collaborative assignments, active learning, and project-based learning created the opportunity for deep social and learning ties to develop between students. Literature from other disciplines is presented below, highlighting findings that show desired network structures can be achieved by design.

Urban spaces are being created specifically to induce creativity and promote consumption habits and connectivity [26]. For example, Neal [27] described how a well-connected network of streets make it easier for people in the community to travel from place to place in contrast to poor quality and unfriendly streets. Similarly, literature from sustainability shows that patterns of interaction (i.e., communication patterns and activities that build collaborative capacity) structure networks, and that relationship patterns in a network have the potential to change outcomes [28, 29]. Research in sustainability has found that, “changing the behavior of one person can change the behaviors of other actors in a social network. Thus, interventions meant to change environmentally relevant behaviors or beliefs may have a social multiplier effect that depends on social network structures” [29] (p. 588). Similarly, another study on LEED certification found that the facilitation practices of teams can impact the outcomes and influence the network [30]. These studies beg the question: If networks can be designed to create networked communities and promote sustainable behaviors, then what is the potential to use network architecture in an applied educational context [4, 5]?

In the field of network analysis, researchers are primarily focused on examining networks that have already been created by interactions in communities, businesses, and other organizations and groups [31]. To date, researchers have seldom written about how networks are structured based on different social interactions [4, 5, 32, 33]. Often, these social interactions are driven or developed by a single person or potentially a two- or three-person team. For this study, we introduce a new term to describe the actor who structures the network–archintor^TM^. An archintor^TM^ (architect + instructor + facilitator) is the individual responsible for being the network architect. These people might be individuals who identify as leaders, problem solvers, facilitators, instructors, and others. Can such actors be the archintor^TM^ whereby they create expectations that generate a specific network structure? Using applied social network analysis in this context allows for a paradigm shift in current education assessment approaches. A paradigm shift results from a change in the process of thinking often due to the introduction of new knowledge or evidence. The result of this shift is a new and different way of thinking about and/or conceptualizing how something should be done, made, or thought about [34].

This study used social network analysis, informal interviews, and course syllabi to provide insights regarding how and why networks form when instructors create implicit or explicit coursework expectations that structure the network. Understanding how the act of explicitly providing coursework expectations can impact the social network that is created can help us understand how information is distributed, explain student interactions, and provide guidelines for increasing the student learning network. All courses have a syllabus with coursework expectations; therefore, we hypothesized that if we understood which coursework expectations created networks conducive to learning, then in practice, instructors could explicitly detail in their syllabus the expectations to socially construct the desired network and to be the network archintor^TM^.

The purpose of this study was to use social network analysis to answer the question: do different instructor coursework expectations change the network structure within a classroom? This overarching research question was explored using four sub-research questions: 1. How do classroom networks change pre- to post-test based on different instructor coursework expectations? 2. How do instructor coursework expectations impact the structure of the communication network? 3. Do different instructor coursework expectations impact the learning network? 4. Are communication and learning networks correlated?

### Course descriptions

Below are detailed descriptions of the university sociology courses that were studied. Each description includes: the level number, number of credit hours, a qualitative description of how students learned and interacted, and average student GPA. Table 1 provides an overview of the coursework expectations and learning activities in each of the courses.

**Table 1:** Instructor coursework expectations.

Course	Course Level	Expectation to Exchange Contact Information with Classmates	Lecture^1^	Active Learning^2^	Collaborative Learning^3^	Community-Based Research *or* Community Partner^4^	Students worked in a team^5^
**Sociology Theory**	300	X	X				
**Research Methods**	100		X	X	X		
**Social Change**	400	X	X	X	X	X	X
**Internship**	400		X	X		Varied	
**Capstone Seminar**	400		X	X	X		
**CBR Capstone**	400	X	X	X	X	X	X

1 The lecture format assumes that listening facilitates the transfer of knowledge [35].

2 Active learning occurs when there is a direct experience and interaction with the intellectual, social, and physical environment [36].

3 Collaborative learning or peer-to-peer learning occurs when students learn from other students [37, 38].

4 There are two defining features of community-based research. First, research is conducted *with* not *on* community partners [39]. Second, students engage in intentional critical reflection about their experience [40].

^5^ Students worked in a single team for the entire semester.

### Sociology theory

Development of Sociology Theory is a three-credit hour, 300-level course for students who have completed one prerequisite course in sociology (Sociology 100 or 105). In this course, students learned about sociology theory from the Enlightenment to the present. This course was a lecture-style class wherein inter-student connections were not required. Hypothetically, a student could pass the class without ever knowing another student in the course. The instructor, however, changed the coursework expectations slightly. During the first week of classes, students were instructed to exchange phone numbers with two other classmates. If they missed class (for any reason) they were expected to reach out and contact those students to see what was missed. In the study, the average class GPA was 3.17, which provides some indication that students largely met the course expectations.

## Research methods

Research Methods was a three credit-hour, 100-level course. In this course, students learned about sociology methods including data gathering, research design problem formulation, and their application to sociological problems. The physical classroom was set up like a traditional-style class where everyone faced the front of the room. However, the instructor had several coursework expectations for students that focused on encouraging student-to-student interactions. On the first day of class, the instructor asked students to ‘get up’ and introduce themselves to someone new. In addition, the teaching style was intentionally interactive with space for comments, questions, and concerns. The instructor frequently had students collaborate with a neighbor during the lecture to answer questions; and finally, students participated in group projects. In the study, the average class GPA was 3.36 which provides some indication that coursework expectations were met.

### Applied Social Change (Social Change)

Applied Social Change is a three-credit hour, 400-level course for students who have completed one prerequisite course in sociology (Sociology 100 or 105). The course was very interactive, wherein students worked to create social change by conducting a community-based research (CBR) project in small groups. There are several distinguishing pedagogical characteristics of CBR. First, the course was facilitated based on the principles of CBR wherein students engage in a large project throughout the semester *with* a community partner [39]. Second, students engage in team activities and write reflections to process their learning [40]. Third, because the research was being conducted *with* the community partner, students had to be responsive to new information, changes in the community, and other variables. During class periods, student desks were often moved to enable and enhance team discussions. In the course, students self-selected into groups. Students put their names and phone numbers on a piece of paper to exchange contact information. A distinguishing feature of the course was that a single grade, worth 25% of the overall course grade, was given to the entire team. In the study, the average class GPA was 3.19, which provides some indication that coursework expectations were largely met.

### Internship

The Internship course is a three-credit hour, 400-level course for students in their junior or senior year. It is one of three capstone course format options for sociology majors. Before students take this course, they must complete several prerequisite courses (SOC 210 or STAT 200 to 499) and (SOC 301 or SOC 302) and (SOC 311) and (SOC 313 or SOC 314 or SOC 315 or CS 110). The primary objective of the internship course was to expose students to real-world experiences. Students worked 150 hours for the local agency that they selected. Internship experiences varied. Some students conducted research, others answered phones, and some filed papers. In addition, they participated in a 1-credit hour seminar. The 1-credit hour seminar met every other week to discuss their internship experiences and other professional development activities. The classroom setup was traditional, wherein students faced the front of the room and listened to a lecture. The social network survey did not provide a space for comments; however, several students from the internship class wrote comments about how they do not meet frequently, they only look at the back of their classmates’ heads, and they do not interact much in class. In the study, the average class GPA was 3.89 which provides some indication that coursework expectations were met.

### Capstone seminar

Capstone Seminar is a three-credit hour, 400-level course for students in their junior or senior year. It is one of three capstone course format options for sociology majors. Before students take this course, they must complete several prerequisites in sociology (SOC 210 or STAT 200 to 499) and (SOC 301 or SOC 302) and (SOC 311) and (SOC 313 or SOC 314 or SOC 315 or CS 110). Students learned how to apply sociological concepts via research projects. The Capstone was designed as a discussion-based seminar wherein students completed semester-long research/capstone projects. Although the students did not work in teams, the course did have two group assignments. For each assignment, students were placed into different groups; thus, students interacted, socialized, and learned from two different sets of classmates. For the first group assignment, students read two books together in small groups of three. For the second group assignment, they were organized into small research groups to conduct independent research on a topic of their choice. Throughout the semester, students gave research updates, and during finals week they presented their research project to the class. In the study, the average class GPA was 3.57 which provides some indication that coursework expectations were met.

### Community Dynamics and Development Capstone (CBR Capstone)

The Community Dynamics and Development Capstone was a four-credit hour, 400-level course, which included a one-hour lab on Friday afternoons. The capstone was for students in their junior or senior year. It is one of three capstone course options for sociology majors. Before students take this course, they must complete several prerequisite courses (SOC 210 or STAT 200 to 499) and (SOC 301 or SOC 302) and (SOC 311) and (SOC 313 or SOC 314 or SOC 315 or CS 110). Similar to the Applied Social Change course, the course was designed around a single CBR project. The research topic and focus varied every year based on the community partner. During the second week of the semester, students self-selected into their research teams, and shared contact information. During class, student desks were often moved to enable and enhance team discussions. Students gave a final presentation that included their data, findings, and products to the community partner. A distinguishing feature of the course was that grades were given to the entire team. There were no individual grades based on the team project which was worth 25% of the course grade. In the study, the average class GPA was 3.47 which provides some indication that coursework expectations were met.

## Methods

The data for this mixed-methods study were collected by conducting informal interviews and administering social network surveys. All data were collected before the 2020 Covid-19 pandemic. Mixed methods were suggested by one study [41] as being the best approach to capture the full extent of student learning outcomes. Further, another study [42] (recommends combining social network analysis and qualitative content analysis for new methodological possibilities. This study extends their methodological recommendations of combining social network analysis with surveys and qualitative data in a comparative study of three course formats. All research was conducted following IRB Protocol #18-7926H which was approved by the Research Integrity and Compliance Review Office (RICRO).

### Qualitative data

Before the survey was administered in each class, the researcher conducted an informal interview with the professor about student interactions and course expectations. The purpose of the interviews was to have a deeper understanding of classroom interactions and coursework expectations to better interpret the social network diagrams. Participants signed interview consent forms approved by RICRO. These field notes were recorded to compare different interactions. After the survey results were analyzed, to increase understanding of interactions, the researcher conducted four qualitative interviews with two students from Sociology Theory and two students from Social Change. The students were recommended by the instructor. The students were asked questions about the course format and Interviews lasted approximately 45–60 minutes. The students discussed the courses, their final projects, their feeling of accomplishment, and their group members (if applicable).

### Social network survey

A social network survey was used to determine how the knowledge networks changed from the start of the semester to the end of the semester in the selected sociology courses.

### Sample

Everyone on the course roster, the instructor, and GTA (when applicable) were given a social network survey. A total of 176 students and eight instructors were surveyed (Table 2). Most participants were juniors and seniors, and the overall course average GPA ranged from 3.17 in Sociology Theory to 3.89 in the Internship course.

**Table 2:** Social network survey sample.

Course	Number of Students							
	Instructors and TAs	Students	Total	Freshman	Sophomore	Junior	Senior	Graduate
**Sociology Theory**	2	40	42	0 (0%)	1 (2%)	16 (38%)	23 (55%)	0 (0%)
**Research Methods**	1	44	45	0 (0%)	0 (0%)	13 (31%)	31 (74%)	0 (0%)
**Social Change**	2	14	16	0(0%)	0 (0%)	4 (10%)	10 (24%)	2 (5%)
**Internship**	1	38	39	0 (0%)	0 (0%)	1 (2%)	37 (88)	0 (0%)
**Capstone Seminar**	1	14	15	0 (0%)	0 (0%)	0 (0%)	14 (33%)	0 (0%)
**CBR Capstone**	1	26	27	0 (0%)	0 (0%)	0 (0%)	26 (62%)	0 (0%)

#### Data collection

The social network survey was administered twice in each course, once during the first two weeks of the semester, and again during the last two weeks of the semester. The survey used a roster format, wherein all students in the course were listed in matrix rows, and the types of communication and social relationships they had with each student were listed in the columns (See pretest survey in S1 Fig and post-test survey in S2 Fig in S1 File). Following RICRO protocol, the researcher read the consent form out loud and all participants were given a copy of the consent form before participating. Given this format, the value given in Table 2 for the number of students in each course corresponds to the number of students on the course roster, and not necessarily the number of students who took the survey. Nevertheless, if a student was not present on the day the survey was administered, the other students in the class still responded to questions regarding their connections to the missing student. Response rate was between 95%-100% in each class.

#### Measures

The items in the survey assessed prior relationships, forms of communication, and learning (See survey in S1 and S2 Figs in S1 File). This study was based on data from seven of the network questions: Who do you recognize? Who are you connected with via social media? Who do you call/text? Who do you connect with via email? Who do you connect with via social media? Who do you learn from? Who learned from you? Several items were combined in the results. The communication network is a combination of who do you text call, email, and connect with via social media; and the learning network is a combination of who did you learn from and who learned from you.

#### Data analysis

The social network data were analyzed using UCInet software programs [43] and diagrams were created using Visone [44]. Nodes in the diagrams were sized by average degree. The average degree for all diagrams was calculated. Average degree is a measure of the number of ties connected to each node and allows for easy comparison between networks [45]. Density measures the ratio of observed edges to the number of possible edges for a given network [46]. A network with high fragmentation has numerous node pairs that are not in contact with other node pairs. In a network with low fragmentation, node pairs can reach most other node pairs [43]. A Pearson Correlation is used to compare networks. A Pearson correlation uses a permutation-based method known as the quadratic assignment procedure (QAP) to obtain a p-value. [47].

#### Structure of the network

**Fig 1:**
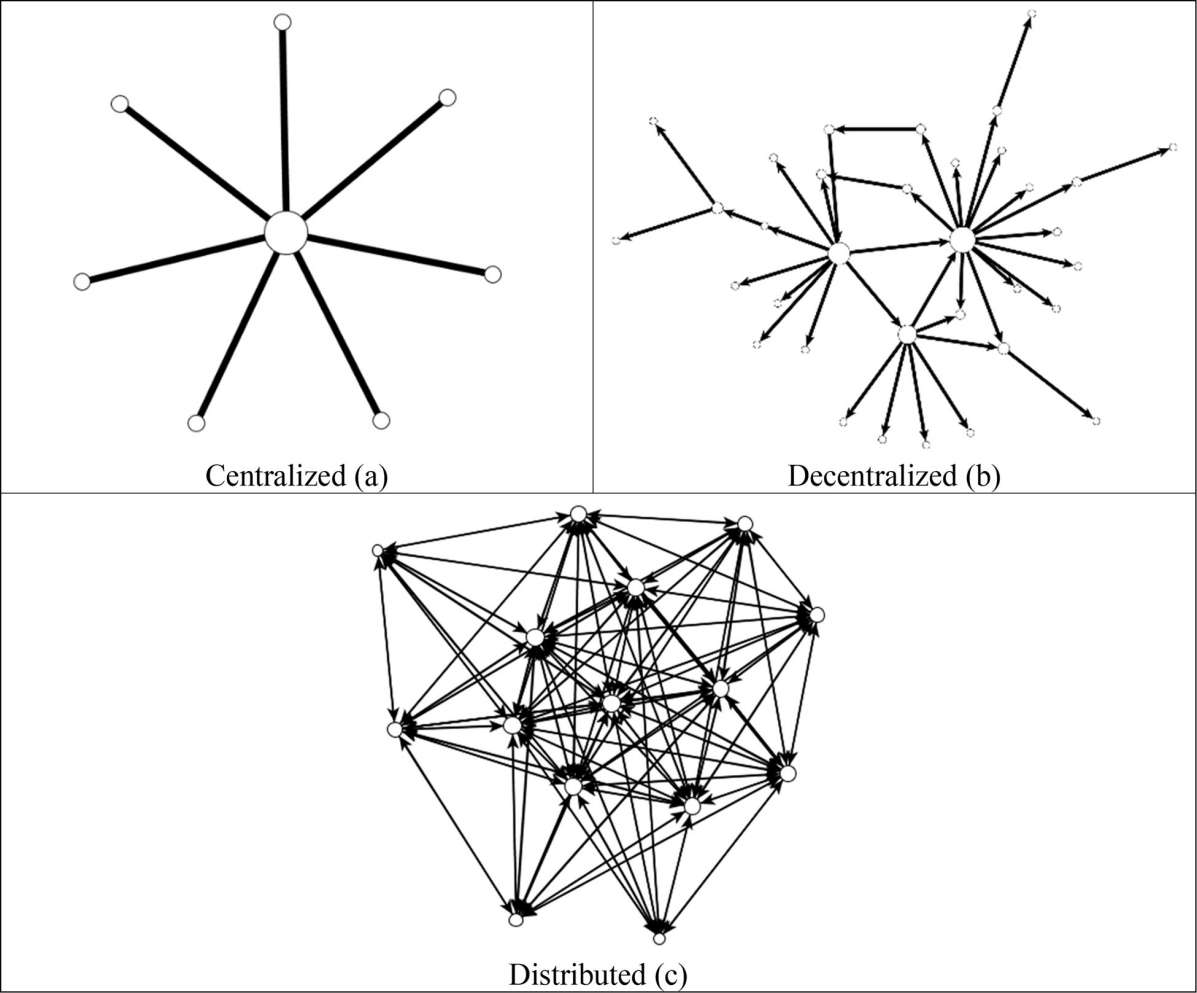
Ideal networks. 1a, 1b, 1c: Idealized depiction of centralized, decentralized, and distributed networks, respectively, modified from Baran [48].

Baran [48] distinguished between three different network structures (see Fig 1). First, a centralized network occurs when all the nodes are centered around a single node or small group of nodes. One study [48] described this network as a vulnerable network because if the central node was removed, the network would fall apart. Therefore, the centralized network is referred to here as a vulnerable network. A decentralized network occurs when multiple nodes serve as key actors in the network. Although it is less vulnerable than a centralized network, if a highly connected node is removed, then the network would have many isolates unable to connect with the rest of the network. Finally, in a distributed network, the nodes have multiple connections with other nodes in the network. This type of network is considered a much less vulnerable network because any node can be removed without the network structure changing appreciably. In comparison, if a peripheral node were removed from the centralized network; the overall network structure would likewise have little to no change; however, if a central node were removed, the network would fall apart.

## Results

The results are divided into four sections to answer the overarching research question: Do different instructor coursework expectations change the network structure within a classroom? The four sections address the four sub-research questions (1) How did classroom networks change pre to post-test based on different instructor coursework expectations? (2) How did instructor coursework expectations impact the structure of the communication network? (3) Did different instructor coursework expectations impact the learning network? (4) Are the communication and learning networks correlated?

### How did the network change during the semester?

**Fig 2:**
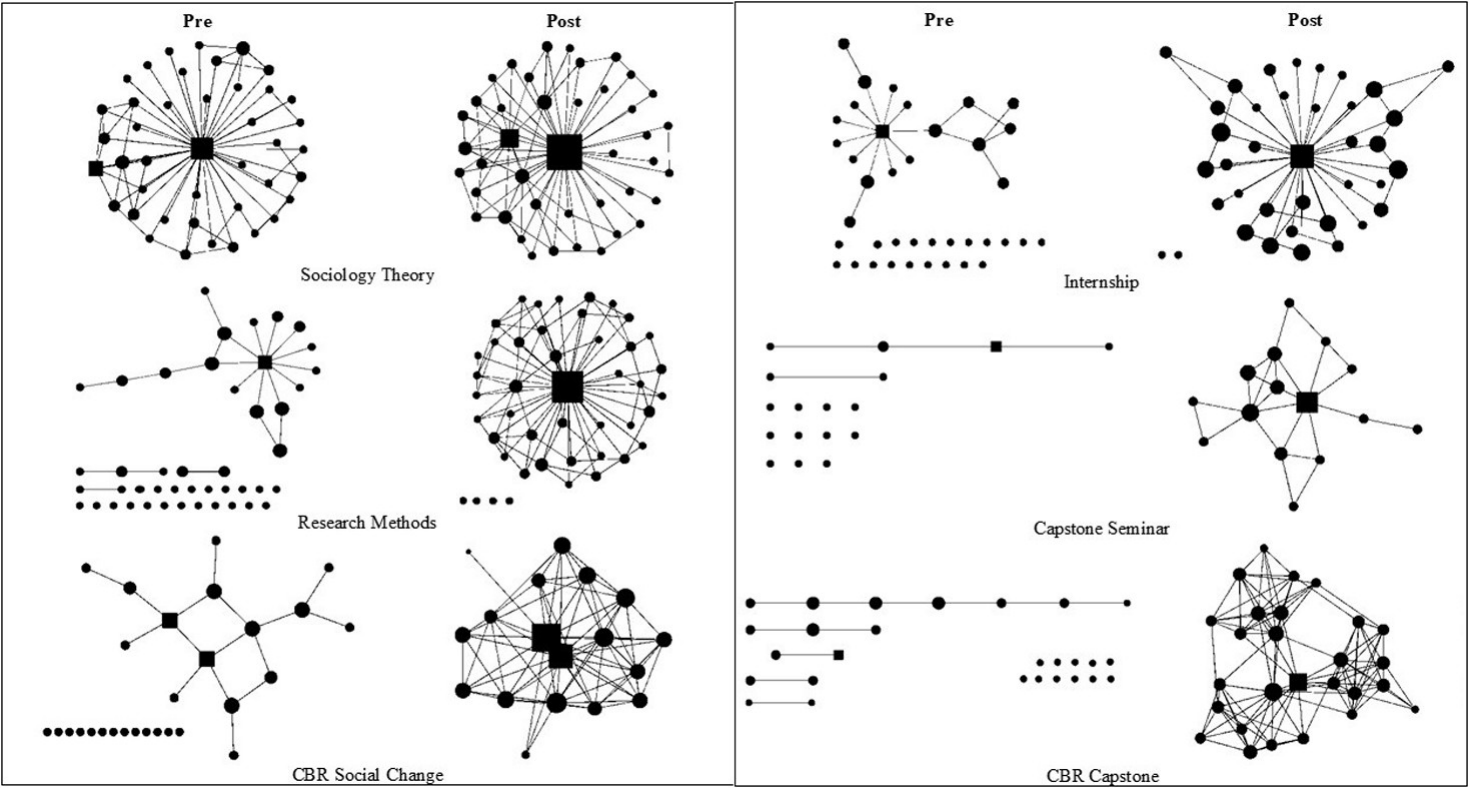
Pre- and post-communication network. The communication network is a combination of three network measures, who do you: email, text, and connect with via social media. Nodes are sized by average degree. Instructors and teaching assistants (TAs) are squares. Students are circles. A circle with no connections is a student isolate. A line between nodes indicates a connection reported on the social network survey.

To develop a baseline of connections in the classroom, the survey asked students who they recognized and who they had class with previously. Students in all the classes recognized, on average, at least one other student in the classroom based on the average degree of the network (Table 3). Notably, students in the Capstone Seminar were all seniors, had taken prerequisite courses in sociology, and yet had the fewest previous connections. Further, students in the lower-level classes, who had taken fewer sociology courses, recognized more of their classmates at the beginning of the semester than did students in the higher-level courses. Fig 2 illustrates how the communication network changed from the beginning of the semester to the end of the semester. There were fewer isolates and more connections for all the classes (Table 4).

**Table 3:** Pre-test network metrics: Previous relationships and connections.

Course	N	Average Degree		Density		Fragmentation	
		Recognize	Social Media	Recognize	Social Media	Recognize	Social Media
**Sociology Theory**	42	4.43	0.29	0.11	0.01	0.26	0.99
**Research Methods**	45	3.31	0.36	0.08	0.01	0.39	0.99
**CBR Social Change**	16	2.33	0.39	0.14	0.02	0.44	0.95
**Internship**	39	2.08	0.31	0.06	0.01	0.68	0.99
**Capstone Seminar**	15	1.00	0.06	0.06	0.00	0.88	0.99
**CBR Capstone**	27	2.89	0.52	0.11	0.02	0.39	0.96

**Table 4:** Social network metrics pre- and post-test communication.

Course	N	Average Degree		Density				Fragmentation	
		Pre	Post		Pre	Post	Pre		Post
**Sociology Theory**	42	2.10	2.45		0.05	0.06	0.59		0.52
**Research Methods**	45	0.75	1.94		0.50	0.13	0.88		0.25
**CBR Social Change**	16	1.00	7.06		0.07	0.47	0.80		0.13
**Internship**	39	0.50	1.50		0.03	0.10	0.97		0.50
**Capstone Seminar**	15	0.18	2.27		0.01	0.16	0.99		0.47
**CBR Capstone**	27	0.67	6.30		0.03	0.24	0.96		0.11

#### Different instructor coursework expectations structure the communication network

Each class in this study had different instructor coursework expectations (Table 1), which led to the sub-research question addressed in this section: *How did instructor coursework expectations impact the structure of the communication network*? In Sociology Theory, all the students were connected to the instructor in the pretest and post-test diagrams, and the network has a similar appearance to the centralized network (Fig 1a). In the course, there was a coursework expectation to exchange phone numbers with two other classmates (Table 1). Evidence in the network diagram suggests that students met this expectation (Fig 2). The average degree in the post network was 2.45, which indicates each student was communicating with 2.45 other students in the classroom (Table 4). This implies that students likely met the expectation of exchanging contact information set during the first week of classes.

Students in Research Methods began the semester with the highest average degree in the recognize (3.31) and class with before networks (3.07) (Table 3). Even though the students recognized each other and had class previously, they were not connected via the communication network. At the beginning of the semester, there is a centralized network (Fig 1A), wherein students were only connected to the instructor, and many students are isolates, where they are not connected to the main network (Fig 2). By the end of the semester, additional connections developed between students in the periphery of the network. The post-network average degree increased from 1.19 to 1.94 which indicates students were communicating on average with approximately two other students in the course. This is an expected increase given the Table 1 course expectations.

In Social Change, students began the semester with few connections to each other and the instructor (Table 3). The coursework expectations were that students worked in small teams to complete a CBR project, and grades were assigned to the whole team (Table 1). At the end of the semester, this network had the highest average degree of all the networks (7.06), was the densest (0.47), and was one of the least fragmented networks, which means nearly all students were connected to the other students in the network. This is the only communication network in the study that had a similar appearance to a classic distributed network (Fig 1C).

The Internship (Capstone) began with few network connections. The coursework expectations were that students complete an internship outside of the classroom (Table 1). Students only attended class for 2-hours every other week. As students were not in class establishing connections and there were no built-in mechanisms for them to make connections outside the classroom, this network ended the semester with the lowest average degree (1.50), one of the lowest densities (0.10), and was highly fragmented (0.50), wherein only half the students could directly reach other students in the network.

The Capstone Seminar began with the fewest network connections (0.18), lowest density (0.01), and highest fragmentation (0.99) (Table 4). The coursework expectations in this small class were to complete two team projects with two different teams. In the post-test, students were communicating with an average of 2.27 classmates, the density increased to 0.16 and the fragmentation was cut in half (0.47). Overall, however, the network remained a centralized network (Fig 1), like those for Sociology Theory and Research Methods (Fig 2). One possible contribution to this is that no team grades were assigned; thus, students’ grades were not dependent on them interacting beyond completing the project.

The CBR Capstone networks had a similar appearance to the Capstone Seminar network at the beginning of the semester. There were very few connections, the density was low, and the network was highly fragmented. The CBR Capstone had similar expectations to the Social Change course, as students worked in three small teams to complete a CBR Research project (Table 1). This resulted in one of the highest average degrees and lowest fragmentation scores in the study (Table 4). In Fig 2, three distinct teams are visible in the network structure which indicates to some extent that students met the expectation of working in teams.

#### Instructor coursework expectations influence the learning network

**Fig 3:**
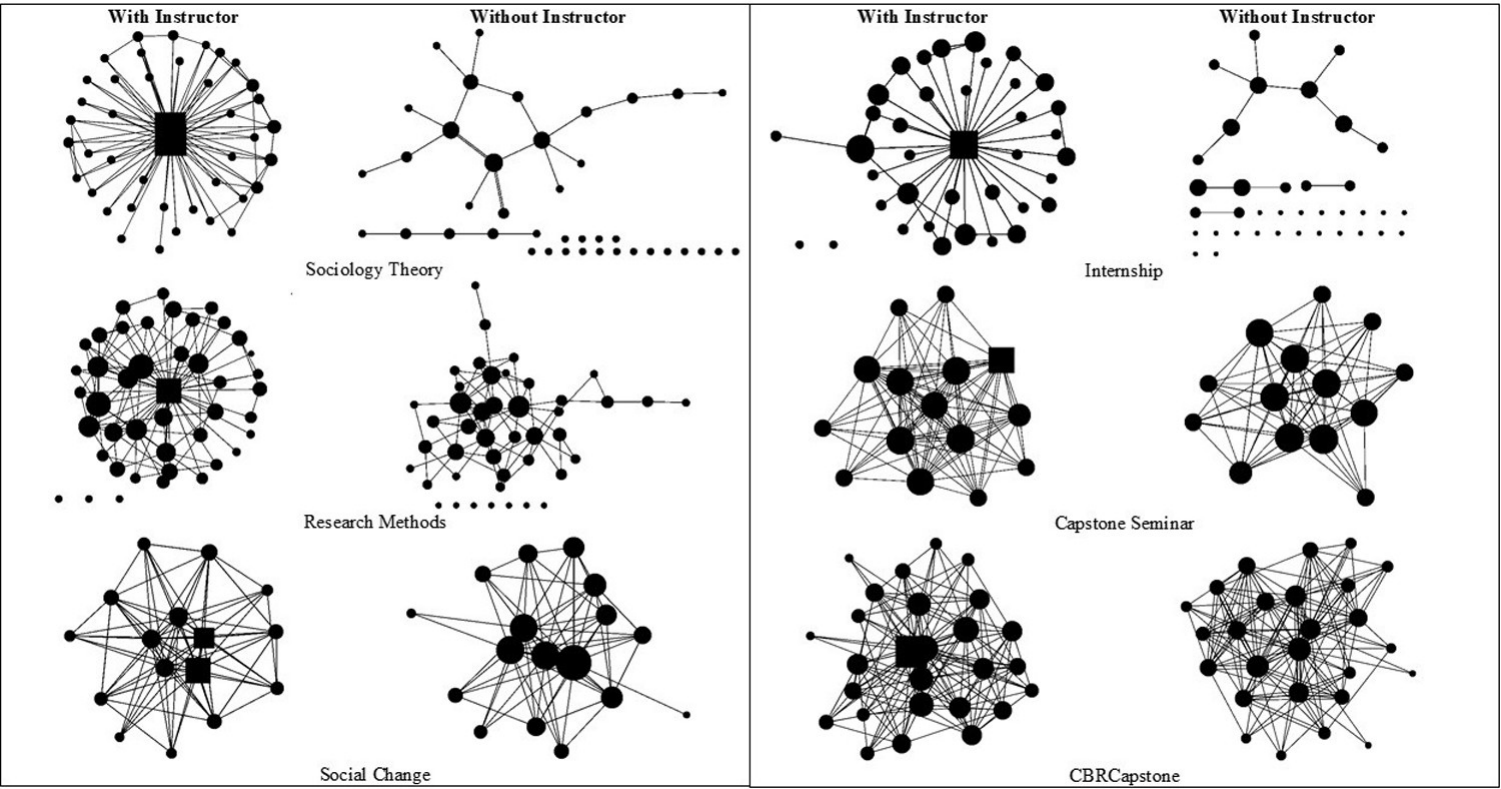
Learning networks with and without the instructor at the end of the semester. Nodes are sized by average degree. Instructors and teaching assistants (TAs) are squares. Students are circles. A circle with no connections is a student isolate. A line between nodes indicates a connection reported on the social network survey.

To address the third sub-research question, *did different instructor coursework expectations impact the learning network*? The networks were analyzed with and without the instructor to understand the impact of the instructor on the architecture of the network. Fig 3 contains the learning networks from the end of the semester for the different courses with (left column) and without (right column) the instructor as part of the analysis.

In Sociology Theory, the instructor and teaching assistant were in the center of the learning network. The network reflects a centralized star-like structure (Fig 3). When the instructor is removed, the isolates outnumber the connected nodes. The average degree in this network dropped from 2.63 to 0.56 without the instructor and fragmentation increased from 0.50 to 0.94 (Table 5). On the social network survey, students in this network did not report that they learned from their peers.

**Table 5:** Social network metrics with and without the instructor learning network.

Course	N	Average Degree		Density		Fragmentation	
		With	Without	With	Without	With	Without
**Sociology Theory**	42	2.63	0.56	0.18	0.04	0.50	0.94
**Research Methods**	45	2.25	0.94	0.15	0.06	0.250	0.83
**CBR Social Change**	16	6.70	3.60	0.38	0.45	0.19	0.46
**Internship**	39	1.00	0.60	0.87	0.00	0.56	0.99
**Capstone Seminar**	15	7.40	6.64	0.53	0.51	0.27	0.43
**CBR Capstone**	27	7.82	6.27	0.30	0.25	0.11	0.23

In Research Methods, the learning network with the instructor had many features of a classic distributed or decentralized network (Fig 1C). Nevertheless, like Sociology Theory, learning was centered around the instructor. Thus, when the instructor was removed from the network, it became fragmented (0.83), and the density dropped to 0.06.

In Social Change, the instructor and TA appear very central in the learning network, yet there are significantly more connections between students. When the instructor was removed, the network largely maintained its original structure. Notably, two student nodes that appear on the periphery when the instructor and TA were in the network moved towards the core of the network without the instructor and TA.

In the Internship, coursework expectations were mostly external to the physical classroom and the others in the classroom (Table 1). This learning network had the lowest average degree (with and without the instructor), and the lowest density and highest fragmentation at the end of the semester. Like Sociology Theory the network falls apart without the instructor.

The Capstone Seminar and CBR Capstone had coursework expectations about team learning, including group projects. In both networks, students reported learning from over seven other students in the classroom. When the instructor was removed, on average, students still reported learning from over six other students in the classroom. Since learning was occurring throughout the network, removal of the instructors did not significantly change the network architecture. The fragmentation values for the Capstone Seminar and CBR Capstone without the instructor were 0.43 and 0.23, which were lower than the fragmentation of Sociology Theory and the Internship with the instructor.

#### Instructor coursework expectations impact communication and learning

The final section of results addresses the question: Are the communication and learning networks correlated? The Pearson correlation between the networks quantifies the degree of concordance between the relationship types (i.e., communication and learning). The Quadratic Assignment Procedure (QAP) was used to assess the statistical significance of the observed correlations. The communication and learning networks were highly correlated for all six courses. This demonstrates an association between the communication and learning networks.

## Discussion

The purpose of this study was to use social network analysis to answer: Do different instructor coursework expectations change the network structure within a classroom? Using social network analysis in this applied context signals a paradigm shift in applied social network analysis. To date, literature is limited regarding the use of social network analysis to determine how networks can be influenced in an applied setting. This study demonstrates that different coursework expectations established by the classroom instructor resulted in different classroom networks. The results of this study further signal the need for a shift in the changing role of the university instructor, and a paradigm shift in social network analysis as a method, educational assessment, and the role of the archintor^TM^ to design classroom expectations to achieve an optimal network structure.

During the Covid-19 pandemic, universities suffered mass reductions in enrollment because students did not believe they would get the college experience [3]. What was missing? Why did students think they would not get the college experience? This study supports a growing body of literature that learning is a social process [11, 12, 49–52], and thus students will not get the college experience during Covid-19 because they will not be learning, building knowledge, and connecting with their peers (and instructors) in the same way. What implications does this have for the post-pandemic world? Our study found that small changes such as exchanging contact information, moving around a classroom to meet a classmate, and group projects positively impacted the average degree and density, and decreased fragmentation in the network. If different classroom expectations influence the network structure, then instructors can set expectations specifically to create networks that are conducive to interactions and learning, effectively serving as the network archintors^TM^. This can also potentially be done in an online environment if instructors are vigilant and ensure students connect and network in meaningful ways.

In a traditional university lecture hall, the professor stands at the front of the classroom and delivers or transfers knowledge to the students. This type of classroom experience creates a star-like network structure (Fig 1A, Centralized network; Fig 3, Sociology Theory), where the professor is the central node in the classroom network and student nodes are in the periphery. In these types of networks, it is the responsibility of the professor to facilitate the classroom activities, expectations, and the potential connections among students in the network. However, adding a few activities like exchanging contact information and learning in small groups can transform the network from star-like to de-centralized (e.g., Fig 3, Research Methods).

In the communication and learning networks, courses that had collaborative learning expectations, like peer-to-peer and collaborative learning (Table 1), resulted in distributed networks at the end of the semester (Fig 3). Courses that did not have collaborative learning expectations resulted in centralized and decentralized networks at the end of the semester, with none achieving a distributed network. In the learning networks (Fig 3), removing the instructor in courses with collaborative learning expectations only changed the structure of the network slightly. Many of these networks maintained a distributed network structure even after the instructor was removed. In contrast, courses that used primarily lecture-based learning activities frequently had more isolates than connected nodes when the instructor was removed. These observations indicate the learning networks in these courses are much more vulnerable and may indicate that the more complex learning beyond simple repeating of information may not be as robust in these environments as it is in those that maintained a distributed network structure.

Three of the courses in the study used collaborative learning techniques: CBR Social change, Capstone Seminar, and CBR Capstone. These coursework expectations created networks with the highest average degrees, most dense networks, and lowest fragmentation. A common characteristic in these three-course descriptions is that students worked in more than one group, or their group worked with more than one group. These conditions created the least vulnerable networks in the study. Notably, although many studies do not differentiate between different types of capstone classes there were three capstone classes in this study (Internship, Capstone Seminar, and CBR Capstone), and their different coursework expectations created different network structures [53]. This aligns with a study which found different long-term outcomes from different capstone class formats [53].

**Fig 4:**
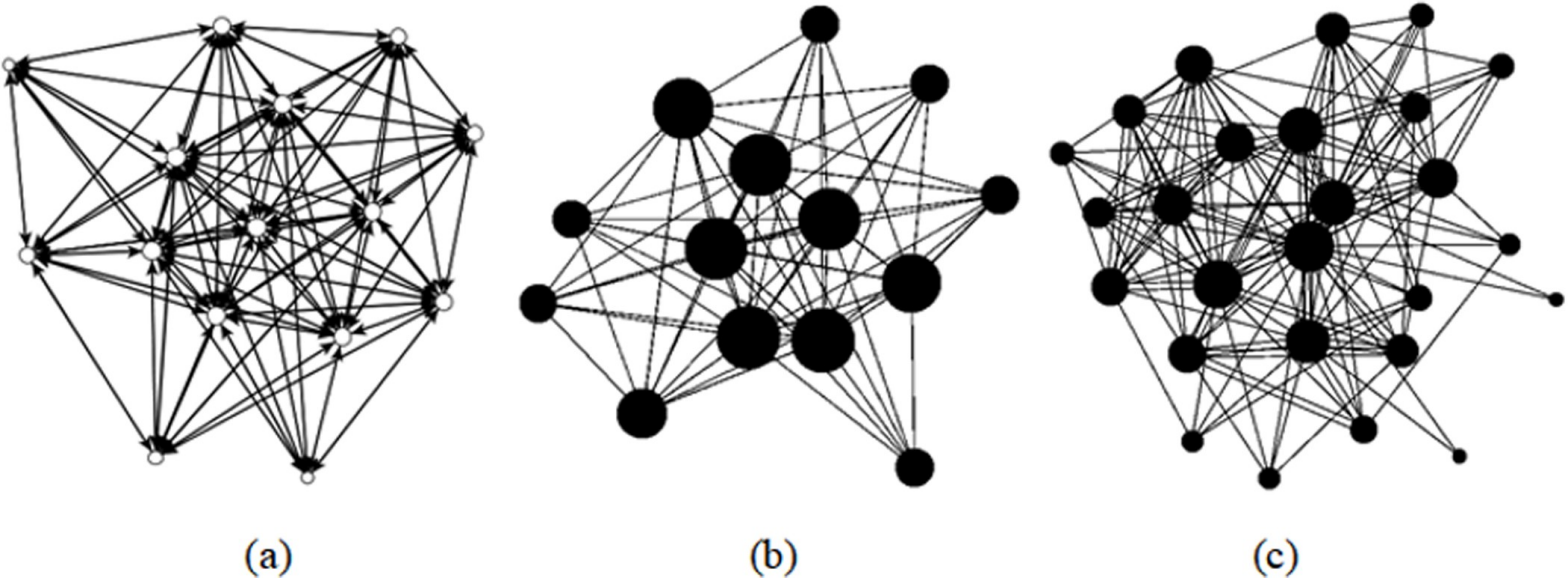
Comparison of (a) ideal distributed network. Networks from Fig 1C and end of the semester learning networks from (Fig B) Capstone Seminar and (Fig C) CBR Capstone courses from Fig 3.

What is the optimal network of a classroom? We propose that it is the distributed network described above in Fig 1C and presented in comparison to other classroom networks created via instructor expectations in Fig 4. Notably, these networks are remarkably similar in terms of the interconnected nature and the observation that all the nodes in each network have two or more connections. For the course learning network diagrams, Fig 4B and 4C, these networks do not contain the instructor or the TA, which makes their “ideal” structure even more significant as these networks evolved based primarily on the instructor’s course expectations.

In a fully connected learning network, everyone’s ideas are contributing to the body of knowledge. Archintors^TM^ can create these types of networks by creating expectations around sharing information, solving problems that require collective cognitive responsibility, and encouraging listening to diverse perspectives. One study [48] described centralized and decentralized networks as vulnerable and the distributed network as less vulnerable. Distributed networks are less vulnerable than the centralized and distributed networks in a learning environment because it would be resilient if a student dopped the class or the instructor missed a day of class. Furthermore, it would promote learning from and understanding of different perspectives. In terms of a learning network, a vulnerable network would likely only provide information from a single source and would not allow room for interpretation, collaboration, and differences of opinion.

We believe one reason students attend large universities and take classes in large lecture classes is so they can be anonymous. Based on our study, however, being anonymous in a classroom does not create a distributed network, and probably negatively impacts deep learning and behavior. For learning to be relevant in the 21^st^ century, graduates must do more than cite facts and figures they have Googled; they must be able to demonstrate the application of knowledge, its relevance and veracity, as well as being able to process information and learn with others.

Recent studies have framed learning as a social process and suggest that social interactions and learning are intertwined [12, 14, 49, 51, 54]. The association between the learning and communication network for all the networks provides additional evidence that learning is a social process. Even in courses with more vulnerable learning networks like Sociology Theory, the networks were associated (Table 6). This association further indicates that the coursework expectations impact the network that develops, and these results add to the growing body of evidence that social interactions and learning are intertwined [31, 55–59]. This suggests that instructors need to engage in ongoing observation, both in person and online, of student academic and social interactions and how each is related to learning.

**Table 6:** Social processes of learning: Communication to learning networks.

Course	N	Correlation	P value
**Sociology Theory**	42	0.72	*P* < 0.0002
**Research Methods**	45	0.70	*P* < 0.0002
**CBR Social Change**	16	0.38	*P* < 0.002
**Internship**	39	0.73	*P* < 0.0002
**Capstone Seminar**	15	0.03	*P* < 0.002
**CBR Capstone**	27	0.50	*P* < 0.0002

### Paradigm shifts

Thomas Kuhn introduced the term “paradigm shift” in a book about scientific revolutions. Kuhn explains that almost every scientific revolution is, at first, a break with old traditions and paradigms [60]. Kuhn explains how paradigm shifts occur. First, there is “normal science” which is the status quo. Second is “extraordinary research” which is characterized by its exploratory nature. Third, is the “adoption of the new paradigm” where a new generation grows-up familiar with the research and it becomes the status quo [61]. From a basic definitional standpoint, a paradigm shift is a fundamental change in approach or underlying assumptions [62]. The literature is rife with publications regarding educational paradigm shifts in the areas of teaching modality, diversity, access to mental health, technology, internationalization, and many others [63–65]. This study signals three paradigm shifts that change the approach and assumptions related to the learning process in classrooms: (1) the practical application of social network analysis as a method; (2) the use of applied social network analysis to study classroom structures; and (3) the need for instructors to become archintors^TM^ that create the most effective classroom structures to advance learning in the 21^st^ century.

The first paradigm shift focuses on the critical need for social network analysis in an applied context to investigate and identify desired network types to support archintors^TM^. Hypothetically, if scientists could identify the desired network for learning, then they could also identify and create the most impactful networks for the university to increase student retention, improve mental health, and more. Are there optimal networks for learning? Are there optimal networks for solving wicked problems, building green buildings, managing a crisis, creating social change, scientific teams, and more? If we know the network structure we want to achieve, then the archintor’s^TM^ task lies in creating expectations for everyday targeted interactions to structure the desired outcome. Currently, most social network analysis is used to test networks after they are already created. Using social network analysis as a method and a practical tool marks the first paradigm shift. Essentially, this shift changes the thinking about social network analysis as a method designed to primarily measure relationships and information flow in classrooms and other spaces to thinking about it as an effective and powerful method that archintors^TM^ can use to create desired and optimal networks [66].

The second paradigm shift comes from the belief that most instructors view learning as a two-way street where they are facilitators of the learning process. Nevertheless, historical patriarchal models wherein the instructors stand at the front of the classroom and lecture to the students still exist in practice. This “talking head” model of transferring information to students and thereby effecting “learning” is a social construct that continues largely because both instructors and students tacitly agree that this is an acceptable form for classroom learning [67]. Arguably, the present study provides yet another demonstration that this method is less effective for developing robust learning outcomes. In contrast, archintors^TM^ plan and prepare relevant course expectations and activities to develop the network structure they want to create in their classroom. Below we provide specific recommendations for archintors^TM^ to create optimal classroom networks.

For the third paradigm shift, the term archintor^TM^ best describes the role of the instructors in the world who are creating course expectations to build well-connected, effective networks. This concept and practice, however, need to be explored further to determine best practices for professional development; a greater understanding of network analysis as a tool to improve student learning and success; and the impact on tenure and other professional advancement structures. In addition, award and recognition mechanisms at universities need to promote and award archintors^TM^ who are intentionally creating fully-connected classroom networks.

Collectively, these shifts have far-reaching implications. In the classroom, understanding the classroom network and the expectations that impact the structure of the network are important for archintors^TM^ to prepare students to enter a workforce that is uncertain and unpredictable in the world today [2, 3]. Methodologically, future research about optimal and desired network structures could help archintors^TM^ solve wicked problems, manage a crisis, create social change, and more.

#### Applicability beyond the classroom

**Fig 5:**
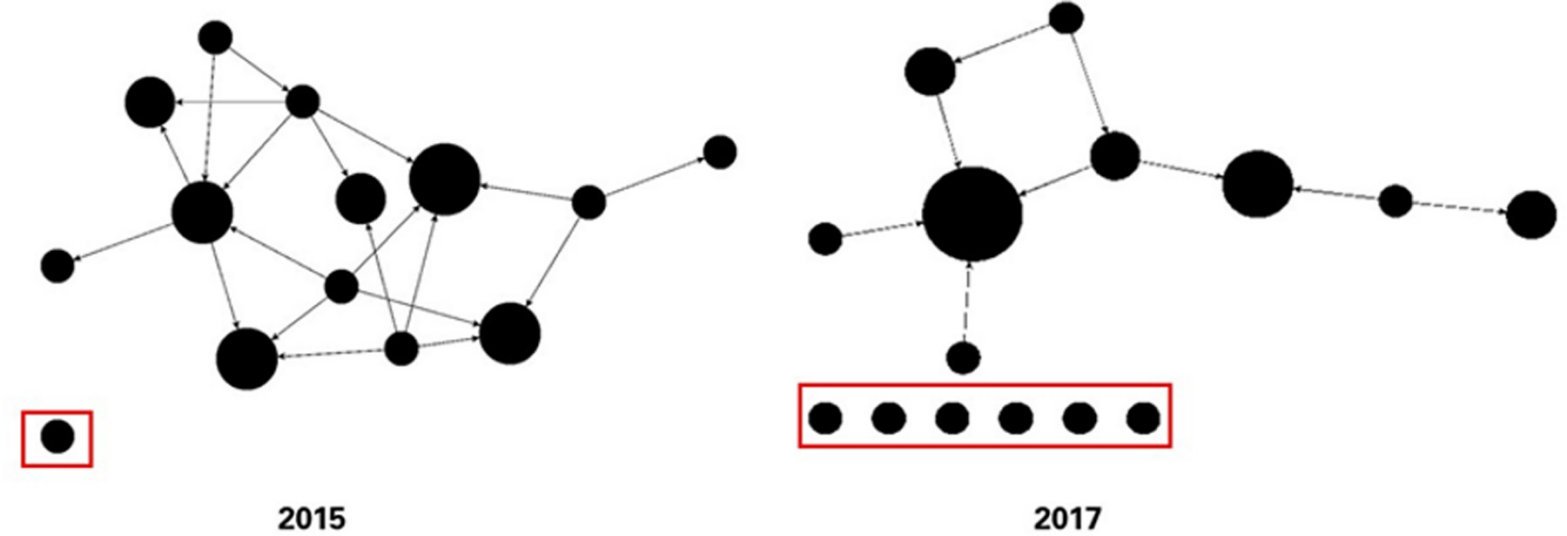
Mentoring network. On this scientific team, data were collected in 2015 before the PI went on a sabbatical and again in 2017, after the sabbatical. The departure left the team without key mentorship connections. Notably, team data collected in 2015 displays a single isolate whereas data collected after the sabbatical indicates six isolates, suggesting the mentees had not re-established connections after their mentor’s return from sabbatical.

This study uses the framework of classrooms because of their relatability across different areas of science. The role of the archintor^TM^, however, goes beyond the classroom, with relevance to all scientific groups and teams. For example, the data in Fig 5 were collected about the mentoring network of a scientific team at two time points (2015 and 2017). Between data collection time points, the PI went on sabbatical with short notice. In 2015, there is only one isolate on the team which indicates that 12 out of the 13 team members were either mentoring or being mentored by other team members. In contrast, the 2017 mentoring network contains six total isolates and fewer overall connections. The network was disrupted by the PI’s departure and had not re-established mentorship connections. An archintor^TM^ could take steps prior to the departure of a key team member, to ensure that team members have other mentors on the team before leaving.

## Limitations

First, this study did not use pre-post learning assessments. It may be important to explore the use of assessments, although under scrutiny in the literature [47, 48, 50], as a strategy to link learning networks to learning outcomes [68–70]. Second, this study only looked at classroom interactions. University instructors and especially student affairs professionals dedicate their lives to connecting students via programs like dormitory-based learning communities, Greek life, alternative spring breaks, welcome events, university traditions and more. The researchers did not ask the students how they connected or when they connected. It is possible that students made connections in and out of the classroom because of other archintors^TM^. Third, the study was only conducted with sociology courses. Future research needs to test different networks in other disciplines and other areas, which may involve additional classroom modalities (e.g., laboratory courses). Fourth, due to the sampling approach, several variables were not considered during this research, such as temporal differences, class size, as well as other confounding factors. Fifth, social network data were collected during multiple semesters and years. Ideally, all data would be collected during the same academic year to avoid any bias introduced by historical effects or policy changes at the university.

### Recommendations

How can you be an archintor^TM^? This study provides further evidence that it is important to explicitly foster communication, learning, and social connections in learning environments. For faculty, instructors, and universities, who want to be the archintor^TM^ in their classrooms, we provide the following recommendations.

For faculty and instructors, first create expectations that support student connections. Small changes such as group projects (Research Methods) changed the learning and communication networks when compared to a traditional lecture-style course. Second, larger differences were identified when there were two group projects (Capstone Seminar) and students worked in teams to complete a CBR project with a community partner (CBR Social Change and CBR Capstone). Group projects need to require communication, sharing of knowledge, and collective cognitive responsibility. If students work independently (rather than infuse and integrate) on the project rather than collaboratively, they will not receive the benefits that are realized by connected and cohesive group members. Third, this is possible in a large lecture hall. Even in the largest lecture hall, it is possible to get to know your neighbor, do a “think, share, pair” exercise that includes exchanging names, participate in a group project, join a study group, exchange contact information and, more.

Learning is a social process, and we recommend students investigate real-world problems together. One model for this is community-based research projects or CBR. In CBR projects, students conduct research with (not on) a community partner. These types of projects require collaboration, coordination, and communication essential to building an ideal network. A second model from the learning sciences literature has theorized that robust learning occurs when students learn to assess their own learning and take responsibility for advancing the learning in groups or teams, called collective cognitive responsibility. The idea is that in a course “students build on one another’s idea contributions and then rise above to find increasingly high-level accounts, helping to create the coherence that drives them toward deeper understanding” [17]. Students take responsibility for the knowledge that’s produced because everyone’s input is needed to build and create new knowledge to solve the problem.

These types of networks cannot be created without strong university-wide support. Universities classrooms need an archintor^TM^ who is invested in creating a network to support the social process of learning. Universities need to provide training, education, resources, rewards, and support for course re-designs that promote collaborative learning. This might include information about how to teach team learning. Also, higher education institutions should consider adding questions to student evaluations regarding classroom interactions and they need to consider and value the interactions that take place outside of the classroom. It may be time for classroom evaluations to include a pre- and post-social network analysis of student interactions and relationships. Finally, promotion and tenure standards should acknowledge the value of classroom network archintors^TM^ who create impactful learning expectations that result in social and academic outcomes.

### Future research

A 2019 article in the Chronicle of Higher Education, “Assessment is an Enormous Waste of Time,” explained that universities continue to measure “learning”, “But what if learning isn’t the most important element in a college education?” [68] (p. 3). Recent studies have found little to no evidence that higher education was teaching learning and critical thinking, [68–70]. The article suggests, “Maybe we should just admit that a four-year, face-to-face college education is a good thing, but we’re not sure why” [68] (p. 6). To continue to make higher education relevant for our changing world, we need a paradigm shift in the teaching and assessment of learning. Higher education needs archintors^TM^ who are building and creating the network, and universities that support the paradigm shift. Based on the data from this study and other literature about student engagement [7, 15, 18, 41] future research needs to continue to explore the connection between student interactions and learning.

Future research must focus on three key areas. First, learning assessments that include social network surveys to understand what connections students made and the impact they had on their lives. Second, future studies would benefit from using longitudinal social network surveys and alumni learning assessments to understand more about the social processes of learning and how coursework expectations impact learning outcomes and successful workforce placements. Finally, future research should extend this study and investigate if there are optimal or ideal networks in other arenas. Hypothetically, if scientists could identify the optimal or ideal network for learning, then they could also identify and create the desired networks for universities to increase retention, improve mental health, and create a highly prepared workforce. The future depends on the vital role of the archintor^TM^ and their ability to create desired networks for learning to solve wicked problems, manage crises, and create social change in an interconnected world faced with extreme challenges.

## Supporting information

S1 File Contains S1 Fig: Pretest Survey & S2 Fig: Post-test Survey. (DOCX)

## References

[1] Pendoley R. Owning the responsibility for learning [Internet]. Age of Awareness. 2019 [cited 2022 Mar 16]. Available from: https://medium.com/age-of-awareness/owning-the-responsibility-for-learning-19a5261d428b

[2] Bouchrika I. 11 top trends in higher education: 2020/2021 data, insights & predictions [Internet]. Research.com. 2020 [cited 2022 Mar 16]. Available from: https://research.com/education/trends-in-higher-education

[3] Baston MA. COVID has spurred four positive changes on campuses (opinion) [Internet]. Inside Higher Ed. 2021 [cited 2022 Mar 16]. Available from: https://www.insidehighered.com/views/2021/11/04/covid-has-spurred-four-positive-changes-campuses-opinion

[4] Love HB. Long term learning outcome of sociology capstone courses at Colorado State University [Internet]. Colorado State University. Libraries. Colorado State University; 2015 [cited 2023 May 22]. Available from: http://hdl.handle.net/10217/166936%0Aoai:dspacetest.library.colostate.edu:10217/166936

[5] Love HB. The Social Process of Knowledge Creation in Science (Doctoral dissertation, Colorado State University). 2019 [cited 2023 Mar 4]; Available from: https://search.proquest.com/openview/529c037dc4daac8cc8c16ec7361a9057/1?pq-origsite=gscholar&cbl=18750&diss=y

[6] Keeling RP. Learning reconsidered 2: A practical guide to implementing a campus-wide focus on the student experience [Internet]. 2nd ed. Learning Reconsidered 2. ACPA ACUHO-I ACUI NACA NACADA NASPA NIRSA; 2006 [cited 2021 Sep 6]. 11–16 p. Available from: www.LearningReconsidered.org.

[7] Kuh GD. Excerpt from: “High-impact educational practices: What they are, who has access to them, and why they matter” [Internet]. 2008 [cited 2019 Aug 30]. Available from: https://secure.aacu.org/PubExcerpts/HIGHIMP.html

[8] Sternberg RJ, Grigorenko EL. Teaching for successful intelligence: Principles, procedures, and practices. Vol. 27, Journal for the Education of the Gifted. SAGE Publications Inc.; 2003. p. 207–28.

[9] Love HB, Cross JE, Fosdick BK, Tofany E, Dickmann EM. Teaching Team Science: The Key to Addressing 21st Century Global Challenges. Small Gr Res. 2022;

[10] Love HB, Valdes-Vasquez R, Olbina S, Cross JE, Ozbek ME. Is cultivating reciprocal learning the gold standard for high impact pedagogies? High Educ Res Dev [Internet]. 2021 [cited 2021 Apr 25]; Available from: https://www.tandfonline.com/doi/abs/10.1080/07294360.2021.1896483

[11] Paavola S, Hakkarainen K. The knowledge creation metaphor—An emergent epistemological approach to learning. Sci Educ [Internet]. 2005 [cited 2018 Mar 13];14(6):535–57. Available from: https://link.springer.com/article/doi: 10.1007/s11191-004-5157-0

[12] Duhigg C. What google learned from its quest to build the perfect team [Internet]. New York Times. 2016 [cited 2017 Dec 2]. Available from: https://www.nytimes.com/2016/02/28/magazine/what-google-learned-from-its-quest-to-build-the-perfect-team.html

[13] Sawyer RK. Group genius: The creative power of collaboration. New York: Basic Books; 2017.

[14] Hakkarainen K. A knowledge-practice perspective on technology-mediated learning. Int J Comput Collab Learn [Internet]. 2009 [cited 2019 Feb 18];4(2):213–31. Available from: www.helsinki.fi/science/networkedlearning

[15] Chickering AW, Gamson ZF. Seven principles for good practice in undergraduate education. Biochem Educ [Internet]. 1989 Jul 1 [cited 2019 Feb 7];17(3):140–1. Available from: http://linkinghub.elsevier.com/retrieve/pii/0307441289900940

[16] The National Association of Student Personnel Administrators and The American College Personnel Association. Learning Reconsidered: A Campu-Wide Fouson on the Student Experience [Internet]. Keeling RP, editor. 2004 [cited 2021 Sep 5]. 1–43 p. Available from: https://www.naspa.org/images/uploads/main/Learning_Reconsidered_Report.pdf

[17] Zhang J, Scardamalia M, Reeve R, Messina R. Designs for collective cognitive responsibility in knowledge-building communities. J Learn Sci [Internet]. 2009 Jan 28 [cited 2018 Jan 21];18(1):7–44. Available from: http://www.tandfonline.com/doi/abs/10.1080/10508400802581676

[18] Kilgo CA, Ezell Sheets JK, Pascarella ET. The link between high-impact practices and student learning: some longitudinal evidence. High Educ [Internet]. 2015 Apr 27 [cited 2019 Apr 27];69(4):509–25. Available from: http://link.springer.com/10.1007/s10734-014-9788-z

[19] Curşeu PL, Janssen SEA, Raab J. Connecting the dots: social network structure, conflict, and group cognitive complexity. High Educ. 2012;63(5):621–9.

[20] Gašević D, Zouaq A, Janzen R. “Choose your classmates, your GPA is at stake!” The association of cross-class social ties and academic performance. Am Behav Sci. 2013;57(10):1460–79.

[21] Tomás-Miquel JV, Expósito-Langa M, Nicolau-Juliá D. The influence of relationship networks on academic performance in higher education: a comparative study between students of a creative and a non-creative discipline. High Educ [Internet]. 2015;71(3):307–22. Available from: 10.1007/s10734-015-9904-8

[22] Saqr M, Alamro A. The role of social network analysis as a learning analytics tool in online problem based learning. BMC Med Educ. 2019;19(1):1–11. 31113441 10.1186/s12909-019-1599-6 PMC6530148

[23] Becheru A, Calota A, Popescu E. Analyzing students’ collaboration patterns in a social learning environment using StudentViz platform. Smart Learn Environ. 2018;5(1).

[24] Dou R, Zwolak JP. Practitioner’s guide to social network analysis: Examining physics anxiety in an active-learning setting. Phys Rev Phys Educ Res [Internet]. 2019;15(2):20105. Available from: 10.1103/PhysRevPhysEducRes.15.020105

[25] Ouyang F. Using Three Social Network Analysis Approaches to Understand Computer-Supported Collaborative Learning. J Educ Comput Res. 2021;59(7):1401–24.

[26] Zukin S. Consuming authenticity: From outposts of difference to means of exclusion. Cult Stud [Internet]. 2008 [cited 2021 Sep 5];22(5):724–48. Available from: https://www.tandfonline.com/doi/abs/10.1080/09502380802245985

[27] Neal ZP. The connected city: How networks are shaping the modern metropolis [Internet]. The Connected City: How Networks are Shaping the Modern Metropolis. Routledge; 2012 [cited 2021 Sep 5]. 1–253 p. Available from: https://www.taylorfrancis.com/books/mono/10.4324/9780203101728/connected-city-zachary-neal

[28] Carlsson L. Policy networks as collective action. Policy Stud J [Internet]. 2000 Aug [cited 2018 Sep 16];28(3):502–20. Available from: http://doi.wiley.com/10.1111/j.1541-0072.2000.tb02045.x

[29] Henry AD, Vollan B. Networks and the challenge of sustainable development [Internet]. SSRN. 2014 [cited 2018 Sep 16]. Available from: www.annualreviews.org

[30] Cross J, Meyer M. Knowledge networks and innovation: how facilitation shapes interaction, network structure, and innovation outcomes. In: Knowledge Networks and Innovation: How Facilitation Shapes Interaction, Network Structure, and Innovation Outcomes. Brighton, England: International Sunbetl Social Network XXXV; 2015.

[31] Phelps C, Heidl R, Wadhwa A, Paris H. Agenda knowledge, networks, and knowledge networks: a review and research. J Manage [Internet]. 2012 Jul 5 [cited 2017 Dec 2];38(4):1115–66. Available from: http://jom.sagepub.com/

[32] Mitrany M, Stokols D. Gauging the Transdisciplinary Qualities and Outcomes of Doctoral Training Programs. [cited 2017 Nov 29]; Available from: http://journals.sagepub.com/doi/pdf/10.1177/0739456x04270368

[33] Love HB, Fosdick BK, Cross JE, Suter M, Egan D, Tofany E, et al. Towards understanding the characteristics of successful and unsuccessful collaborations: a case-based team science study. Humanit Soc Sci Commun. 2022;9(1):1–11.

[34] Lombrozo T. What Is A Paradigm Shift, Anyway? [Internet]. 2016. Cosmos And Culture: National Public Radio; 2016 [cited 2023 May 18]. Available from: https://www.npr.org/sections/13.7/2016/07/18/486487713/what-is-a-paradigm-shift-anyway

[35] Research Brew A. and teaching: changing relationships in a changing context. Stud High Educ [Internet]. 1999 [cited 2018 Sep 30];24(3):291–301. Available from: http://www.tandfonline.com/action/journalInformation?journalCode=cshe20

[36] Edwards S. Active learning in the middle grades. Middle Sch J. 2015;46(5):26–32.

[37] Kandlbinder P. Signature concepts of key researchers in North American higher education teaching and learning. High Educ [Internet]. 2015;69(6):243–55. Available from: 10.1007/s10734-014-9772-7

[38] Gokhale A. Collaborative learning enhances critical thinking. 1995 [cited 2022 Mar 5]; Available from: https://scholar.lib.vt.edu/ejournals/JTE/v7n1/gokhale.jte-v7n1.html?ref=

[39] Strand K, Cutforth N, Stoecker R, Marullo S. Community-based research and higher education: Principles and practices. John Wiley & Sons; 2003.

[40] Mitstfer DI, Council for the Advancement of Standards in Higher Education CS. CAS professional standards for higher education. 8th ed.. Mitstifer DI, editor. Professional standards for higher education. Washington, DC: Council for the Advancement of Standards in Higher Education; 2012.

[41] Brownell JE, Swaner LE. High-impact practices: Applying the learning outcomes literature to the development of successful campus programs. Assoc Am Coll Univ PEER Rev [Internet]. 2009 [cited 2019 Apr 7];26–30. Available from: http://search.proquest.com/openview/ddba5447b97a1ae0a446d14740206ad6/1?pq-origsite=gscholar&cbl=26636

[42] Lipponen L, Rahikainen M, Lallimo J, Hakkarainen K. Patterns of participation and discourse in elementary students’ computer-supported collaborative learning. Learn Instr [Internet]. 2003 [cited 2019 Feb 18];13:487–509. Available from: www.elsevier.com/locate/learninstruc

[43] Borgatti SP, Everett MG, Freeman LC. UCINET [Internet]. Encyclopedia of Social Network Analysis and Mining. New York, NY: Springer New York; 2014 [cited 2019 Feb 20]. p. 2261–7. Available from: http://link.springer.com/10.1007/978-1-4614-6170-8_316

[44] Brandes U, Wagner D. Analysis and Visualization of Social Networks. In Springer, Berlin, Heidelberg; 2011 [cited 2019 Mar 13]. p. 321–40. Available from: http://link.springer.com/10.1007/978-3-642-18638-7_15

[45] Giuffre K. Communities and networks: using social network analysis to rethink urban and community studies. 1st ed. Cambridge MA: Polity Press; 2013. 224 p.

[46] Frey BB. Network density. In: The SAGE Encyclopedia of Educational Research, Measurement, and Evaluation. SAGE Publications, Inc.; 2018.

[47] Hanneman RA, Riddle M. Introduction to social network methods [Internet]. University of California, Riverside, CA; 2005 [cited 2018 Sep 16]. Available from: http://faculty.ucr.edu/∼hanneman/nettext/

[48] Baran P. On distributed communications: summary overview [Internet]. On Distributed Communications: Summary Overview. RAND Corporation; 2018 [cited 2019 Feb 19]. Available from: https://www.rand.org/pubs/research_memoranda/RM3767.html

[49] Csikszentmihalyi M. Implications of a systems perspective for the study of creativity. In: Sternberg RJ, editor. Handbook of creativity. Cambridge, England: Cambridge University Press; 1999. p. 313–35.

[50] Ulibarri N, Cravens A, Kernbach S, Nabergoj A, Royalty A. Creativity in Research. Cambridge, England: Cambridge University Press; 2019. p. 1–317.

[51] Love HB, Cross JE, Fosdick B, Crooks KR, VandeWoude S, Fisher ER. Interpersonal relationships drive successful team science: an exemplary case-based study. Humanit Soc Sci Commun [Internet]. 2021 May 6 [cited 2021 Aug 6];8(1):1–10. Available from: https://www.nature.com/articles/s41599-021-00789-8

[52] Sawyer RK. Emergence in creativity and development. In: Sawyer V, John-Steiner S S. M, Sternberg DH, Wakamura J. F et al., editors. Creativity and development. Oxford, England: Oxford University Press; 2003. p. 12–60.

[53] Love HB, MacIlroy K. A comparison of three capstones: survey results from sociology alumni. Teach Sociol. 2021;49(4):360–71.

[54] Cravens AE, Jones MS, Ngai C, Zarestky J, Love HB. Science facilitation: navigating the intersection of intellectual and interpersonal expertise in scientific collaboration. Humanit Soc Sci Commun [Internet]. 2022 Aug 5 [cited 2022 Aug 10];9(1):1–13. Available from: https://www.nature.com/articles/s41599-022-01217-1

[55] Boix Mansilla V, Lamont M, Sato K. Shared cognitive emotional interactional platforms: markers and conditions for successful interdisciplinary collaborations. Sci Technol Hum Values. 2016;41(4):571–612.

[56] Bozeman B, Fay D, Slade CP. Research collaboration in universities and academic entrepreneurship: The-state-of-the-art [Internet]. Vol. 38, Journal of Technology Transfer. Springer US; 2013 [cited 2018 Feb 3]. p. 1–67. Available from: http://link.springer.com/10.1007/s10961-012-9281-8

[57] Hall KL, Vogel AL, Croyle RT. Strategies for Team Science Success: Handbook of Evidence-Based Principles for Cross-Disciplinary Science and Practical Lessons Learned from Health Researchers. Switzerland: Springer Nature; 2019.

[58] Lee S, Bozeman B. The impact of research collaboration on scientific productivity. Soc Stud Sci [Internet]. 2005 Oct 1 [cited 2013 May 22];35(5):673–702. Available from: http://sss.sagepub.com

[59] Zhang HH, Ding C, Schutte NS, Li R. How team emotional intelligence connects to task performance: A network approach. Small Gr Res. 2020;51(4):492–516.

[60] Covey SR. The 7 Habits of Highly Effective People: Powerful Lessons in Personal Change and Habits. Simon & Schuster. 2017.

[61] Kuhn TS (Thomas S. The structure of scientific revolutions. 3rd ed. The structure of scientific revolutions. Chicago, IL: University of Chicago Press; 1996.

[62] Blessinger P, Reshef S, Sengupta E. The shifting paradigm of higher education [Internet]. University World News The Global Window on Higher Education: Penn Graduate School of Education. 2018 [cited 2023 Feb 7]. p. 1–4. Available from: https://www.universityworldnews.com/post.php?story=20181003100607371

[63] Fazel M, Hoagwood K. School mental health: integrating young people’s voices to shift the paradigm [Internet]. Vol. 5, The Lancet Child and Adolescent Health. Elsevier B.V.; 2021 [cited 2023 Feb 7]. p. 156–7. Available from: http://www.thelancet.com/article/S2352464220303886/fulltext 33484659 10.1016/S2352-4642(20)30388-6

[64] Misiejuk K, Ness IJ, Gray R, Wasson B. Changes in online course designs: Before, during, and after the pandemic. Front Educ [Internet]. 2023 Jan 24 [cited 2023 Feb 7];7:1045. Available from: https://www.frontiersin.org/articles/10.3389/feduc.2022.996006/full

[65] Tadesse T, Gillies RM, Manathunga C. Shifting the instructional paradigm in higher education classrooms in Ethiopia: What happens when we use cooperative learning pedagogies more seriously? Int J Educ Res. 2020 Jan 1;99:101509.

[66] Grunspan DZ, Wiggins BL, Goodreau SM. Understanding classrooms through social network analysis: A primer for social network analysis in education research. CBE Life Sci Educ. 2014 Jun 2;13(2):167–78. doi: 10.1187/cbe.13-08-0162 26086650 PMC4041496

[67] Brigati J. Student Attitudes toward Active Learning vs. Lecture in Cell Biology Instruction. Am Biol Teach. 2018;80(8):584–91.

[68] Gilbert E. Assessment is an Enormous waste of time. The Chronicle of Higher Education [Internet]. 2019 [cited 2021 Sep 6]; Available from: https://www.chronicle.com/article/assessment-is-an-enormous-waste-of-time/

[69] Arum R, Roksa J. Academically adrift: limited learning on college campuses. 2011 [cited 2019 Feb 11];259. Available from: https://ebookcentral.proquest.com/lib/csu/detail.action?docID=648124

[70] Sullivan DF, McConnell KD. It’s the assignments—A ubiquitous and inexpensive strategy to significantly improve higher-order learning. Chang Mag High Learn [Internet]. 2018 Sep 3 [cited 2021 Sep 6];50(5):16–23. Available from: https://www.tandfonline.com/doi/abs/10.1080/00091383.2018.1510257

