# Supplementary material for: What is an “ArchintorTM?” A paradigm shift in teaching, facilitation, and learning: The impact of different types of coursework expectations on classroom network structures
Source: PLoS One. 2023 Jul 19;18(7):e0288136. doi: 10.1371/journal.pone.0288136 (PMC10355462; doi:10.1371/journal.pone.0288136)
Supplement: S1 File — (DOCX) [file pone.0288136.s001.docx]

**Supplementary Figure 1: Pretest Survey**

The pretest social network survey was distributed to participants.

| **Class Roster** | **Do you recognize this student outside of class** | **Have you had a class before with this student?** | **Do you connect with this student via social media** | **Do you email this student?** | **Do you have their phone number to text/ call?** | **Do you socialize out with this student outside of class *infrequently* (less than once per month)?** | **Do you socialize with this student outside of class *regularly* (once per month or more)?** |
| --- | --- | --- | --- | --- | --- | --- | --- |
| Professor | Yes | Yes | Yes | Yes | Yes | Yes | Yes |
| Teaching Assistant | Yes | Yes | Yes | Yes | Yes | Yes | Yes |
| Student #1 | Yes | Yes | Yes | Yes | Yes | Yes | Yes |
| Student #2 | Yes | Yes | Yes | Yes | Yes | Yes | Yes |
| Student #3 | Yes | Yes | Yes | Yes | Yes | Yes | Yes |

**Supplementary Figure 2: Post-test Survey**

The post-test social network survey was distributed to participants.

| **Class Roster** | **Do you connect with this student via social media** | **Do you email this student?** | **Do you have their phone number to text/ call?** | **Do you socialize out with this student outside of class *infrequently* (less than once per month)?** | **Do you socialize with this student outside of class *regularly* (once per month or more)?** | **Who did you learn from?** | **Who do you think learned from you?** |
| --- | --- | --- | --- | --- | --- | --- | --- |
| Professor | Yes | Yes | Yes | Yes | Yes | Yes | Yes |
| Teaching Assistant | Yes | Yes | Yes | Yes | Yes | Yes | Yes |
| Student #1 | Yes | Yes | Yes | Yes | Yes | Yes | Yes |
| Student #2 | Yes | Yes | Yes | Yes | Yes | Yes | Yes |
| Student #3 | Yes | Yes | Yes | Yes | Yes | Yes | Yes |
